# A nature-based intervention for bereaved friend and family cancer caregivers: a study protocol

**DOI:** 10.3389/fpubh.2026.1844557

**Published:** 2026-07-15

**Authors:** Rebecca Lehto, Arienne Patano, Zhehui Luo, Jason Moser, Esra’a Sawalmeh, Gwen Wyatt

**Affiliations:** 1 College of Nursing, Michigan State University, East Lansing, MI, United States; 2 Department of Epidemiology and Biostatistics, Michigan State University, East Lansing, MI, United States; 3 Department of Psychology, Michigan State University, East Lansing, MI, United States

**Keywords:** bereavement, cancer, caregiver, digital mental health, grief intervention, meditation, nature, end-of-life

## Abstract

Friend and/or family caregivers (FCGs) of those with terminal cancer are heavily relied upon to address the emotional and physical needs of the patient facing death. Although increasing research has focused on testing supportive interventions for FCGs who deliver home-based care at end of life, limited attention has targeted strategies that support grief processing for bereaved FCGs during the months following the patients’ death. Both natural environments and meditation practices are shown to support directed attention, reflection, and the capacity to remain present with painful thoughts and emotions that are essential for grief processing. This mixed-method study assesses the potential of a new tailored online audio nature-meditative intervention to support grief recovery and healing among 70 bereaved cancer caregivers who are in the first year of bereavement. A primary goal is to assess the acceptability and feasibility of both content and delivery methods. Measures for the 6-week intervention include numbers eligible vs. consented; numbers consented vs. completed; number of weeks using the intervention, and self-report feasibility/acceptability. Potential effect size estimate data for future larger scale work will be collected at three time points: baseline (Time 1; study week 0), at the intervention end (Time 2; week 6), and at follow-up (Time 3; study week 12) on quality of life, bereavement (grief), directed attention, anxiety and depressive symptoms. Additionally, semi-structured interviews with a diverse representative subsample of 15 caregivers will be conducted at the end of the 12-week study to further assess acceptability and feasibility in more depth. Data analyses will include descriptive statistics, linear mixed models, and content analysis. Findings will be used to assess and enhance the intervention prior to wider scale testing with the long-term goal of establishing an evidence-based intervention for supporting bereaved cancer caregivers as they process grief and adapt to their loss.

## 1 Introduction

The number of patients with advanced cancers who die at home continues to grow in the United States (1). Parallel with this growth is the expanded reliance on friend and/or family caregivers (FCGs) who provide supportive care to patients facing death (2–5). While research evaluating support for cancer FCGs has grown substantively, particularly for FCGs of patients receiving active anti-cancer therapies and for those with advanced cancer at the end of life, comparatively limited research has focused on supporting the bereaved FCG post-patient death (6, 7).

Caring for patients with advanced cancer at the end of life is associated with a high psychological burden (2, 8, 9). FCGs often have limited preparation for the multiple challenges they encounter during the progressive decline in their patient confronting death, often putting personal needs aside as they support the patient during the dying process (10–12). The post-death bereavement period can then be laden with the additional distress associated with confronting the loss of a beloved person, loneliness, and stretches of time that were once consumed with caregiving (13–15). Grief is a painful yet natural and universal human response to loss that usually abates with time (16, 17). However, for those FCGs with poorer mental health, there is heightened risk for complicated grief (17). When not addressed, such grief persists over time, increasing depressive and anxiety symptoms, compromising directed attention and cognitive capacity, and adversely impacting quality of life (QOL) while also increasing risk for morbidity and mortality (6, 13, 16). The recent Diagnostic and Treatment Manual (DSM-5-TR) and International Classification of Diseases (ICD-11) addition of prolonged grief disorder (PGD), characterized by intense anguish and impaired functioning 6 to 12 months and beyond post-loss, confirm the importance of addressing bereavement adaptation early in the post-death period (6, 16–19). Factors associated with increasing complicated grief risk during bereavement include female sex, lower income and education level, comorbid mental and physical health conditions, relationship characteristics, inadequate care provision of the patient at end of life and death circumstances, conflict, and burnout (13, 16).

While the needs of specific individuals facing grief vary, clinical bereavement programs provide services such as informational support, psychoeducation, support groups, brief psychotherapy, pharmacotherapy, and individualized counseling with high levels of variability in types, focus, duration, and outcomes across programs offered (20, 21). Several theoretical perspectives have been adopted that address grief recovery. The dual processing framework identifies grief as a dynamic and adaptive process that oscillates between loss-oriented (acknowledging/processing emotional impact; avoidance and denial) and restoration-oriented (life changes/distraction) coping (22). The stress-coping model frames bereavement as a stressor with problem-focused coping devoted to management of the stressor and emotion-focused coping oriented towards grief processing. Other models include Horowitz’s stress trauma framework which addresses the cognitive intrusions and avoidance that are part of the trauma grief experience and the two-track bereavement model which frame biopsychosocial outcomes and loss transformation as separate tracks in grief recovery (23). A more recent theoretical adaptation integrates the two track and dual processing models (23). Grief focused cognitive-behavioral therapies (GF-CBT), meaning centered therapies, peer support, and integrated therapies that combine acceptance and mindfulness-based therapies with CBT have been evaluated for adults with PGD, and in spousal caregivers with positive impact on grief recovery outcomes (24–30). Both face-face and internet delivered GF-CBT are shown efficacious for depression and grief symptoms associated with PGD (29, 31–33). Gaps, however, remain related to sample homogeneity with limited representation of cultural and racial-ethnically diverse groups, evidence implementation challenges due to financial cost, training needs, and travel needs to on-site venues. Interventions are needed for bereaved FCGs that are readily accessible, convenient, and low cost.

A growing body of evidence supports the benefits of meditation as a mind–body practice that strengthens the capacity to stay present with difficult inner experiences (30, 34–36). Regular meditative practices contribute to changes in functional brain activity in the fronto-parietal regions that directly affect the self-regulatory capacity essential for managing stressors such as the difficulties associated with bereavement (34, 37, 38). With consistency and effort, meditative practices strengthen directed attention, also referred to as the cognitive capacity to focus and concentrate, enhancing present-moment interoceptive awareness of current mental and physical events, and support ‘letting go’ of rumination and worries (34, 38). Importantly, as an evidence-based strategy, research that examines its value in supporting FCGs during the bereavement period is essential.

The natural environment provides a calming space for alleviating distress (39–41). Growing research cites nature’s serene qualities that promote relaxation, help manage stress, and improve emotional well-being (34, 42). Research has also demonstrated that natural environment promotes the restoration of directed attention capacity and thus boost effective cognitive functioning (43, 44). Although the formal testing of nature-based interventions for cancer FCGs is a gap in the science (45), those that have evaluated nature-based interventions in adult populations have identified the aforementioned benefits (46).

The provision of low-cost self-care resources that address bereaved FCGs’ QOL and emotional health post-patient death from cancer is both a critical need and a research gap (6, 10). The present study is novel because it assesses a newly developed nature-based healing meditation (NBHM) intervention that blends meditative practices with nature imagery in an on-line format to promote individualized grief processing among bereaved cancer FCGs. Importantly, on-line delivery of psychotherapeutic interventions have grown with demonstrated efficacy in the past two decades (47). There is growing research that has tested both meditative practices and nature interventions via virtual and online formats (36, 48–51) demonstrating an evidence-base that is complementary given interrelated components such as the ‘meditative qualities and reflection opportunity’ often associated with nature. Thus, combining meditation with exposure to nature as an integrated intervention is posited to yield superior benefits to either alone (52). The six-week technology-mediated intervention described in more detail in the methods section offers auditory guided nature-meditation modules that are anchored in six different natural settings (spring meadow, fall meadow, wooded forest, beach scene, solar system/night sky, evening sky), from which participants may select. Importantly, integration of grief adaptation theory, mechanisms underlying meditative practices that promote mindful awareness, and attention restoration theory provides a conceptual avenue for producing an intervention able to support bereaved FCGs.

**Figure 1:**
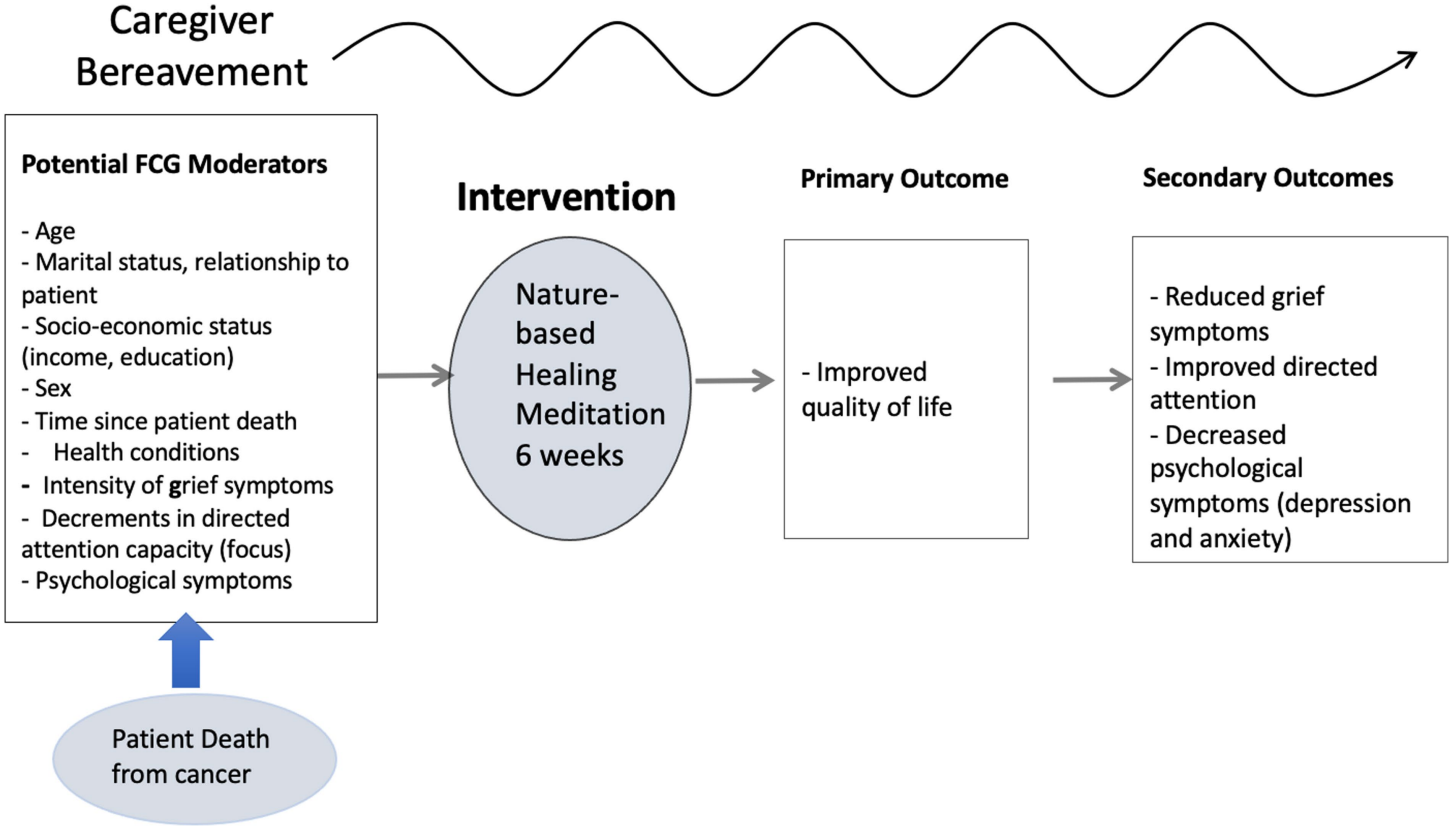
The conceptual framework whereby the NBHM intervention is theorized to support the bereavement process and enhance QOL as the primary outcome. Potential moderators include sociodemographic characteristics, time since the patients’ death, relationship status, pre-existing health conditions, intensity of grief symptoms, directed attention functioning, and psychological symptoms at baseline. By supporting directed attention, grief processing, and psychological health, the NBHM is posited to promote QOL during a time of great challenge and vulnerability.

The purpose of this paper is to describe the research protocol for assessment of a newly developed nature-based meditation intervention for bereaved FCGs of patients who died from cancer. The research aims include: (1) evaluate the acceptability and feasibility of the content and delivery of the NBHM intervention for use by bereaved cancer FCGs; and (2) assess the potential of the NBHM intervention towards improving parameters of bereavement recovery (see Figure 1).

## 2 Materials and methods

### 2.1 Study design

**Figure 2:**
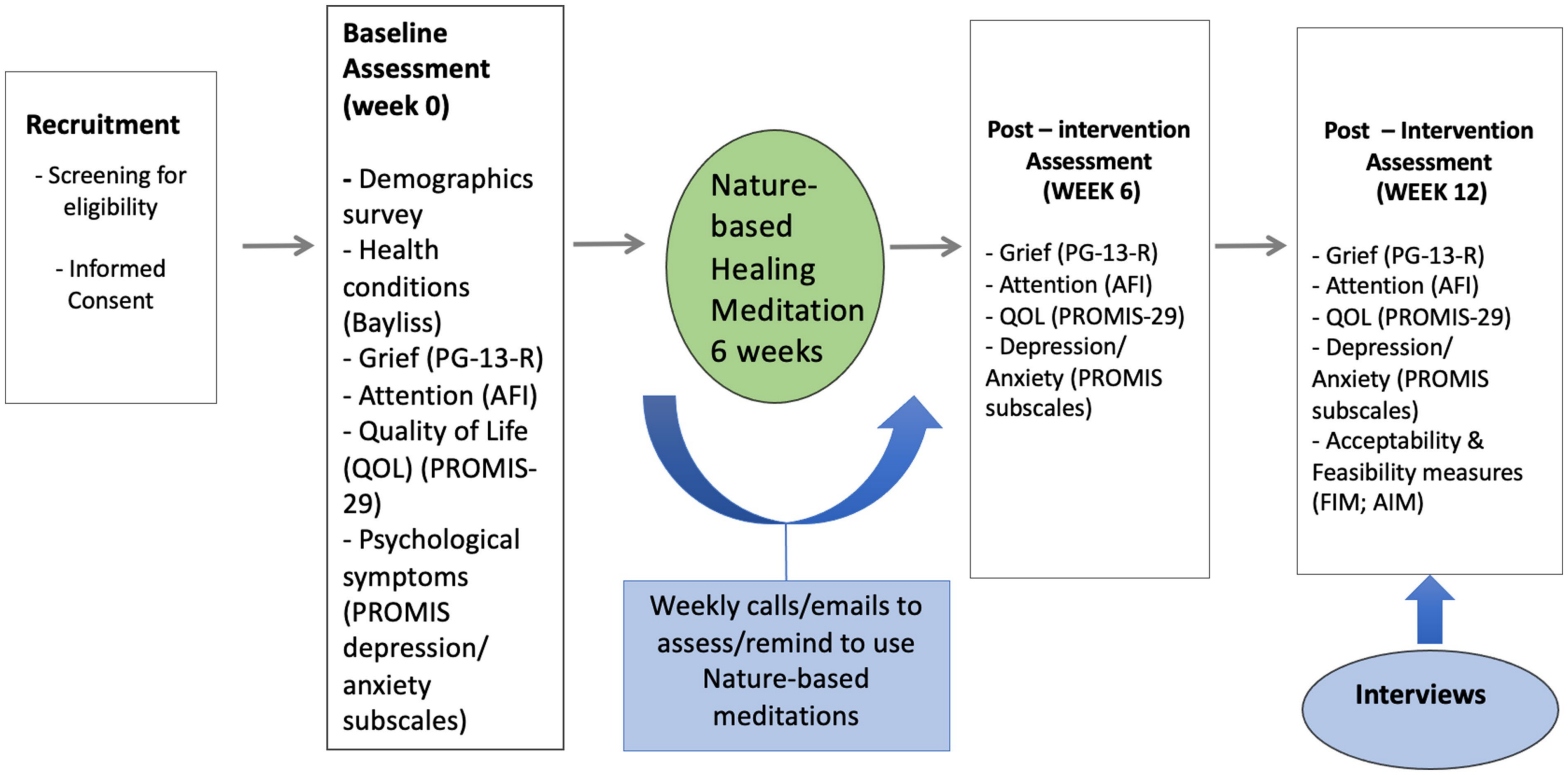
A study schematic including the time points when data collection occurs.

The study uses a single-group, prospective pre-post mixed-methods design to assess the acceptability and feasibility and to estimate effect sizes of the newly developed NBHM intervention on the primary outcome of QOL and secondary outcomes of grief processing (intensity/severity of grief symptoms, directed attention and psychological symptoms). The mixed-method approach will yield important information from both survey and qualitative data regarding the acceptability and feasibility of the NBHM intervention. No comparator is used given the study’s primary aim is to evaluate the feasibility of the new intervention (Figure 2).

### 2.2 Sample

The study sample will consist of adult FCGs. Inclusion criteria are as follows: (1) > 18 years of age; (2) caregiver of a patient who died from cancer during the past one year; (3) able to understand, speak, and read English; (4) able to hear conversation at a normal speaking volume; (5) have a phone and/or an email address where they can be reached; and (6) possess or have access to an internet-connected computer and/or a mobile device capable of displaying web pages. Exclusion criteria are (1) FCGs of persons who died of conditions other than cancer; and (2) FCGs of a patient who died from cancer more than 1 year ago.

### 2.3 Procedures

Following IRB approval, convenience sampling will be used to recruit participants. Study eligibility will ultimately be determined by the project manager following evaluation of on-line eligibility responses. Recruitment will occur in a multi-pronged strategy: (1) study flyers will be posted at community sites including areas where support groups are held (hospice, campus); (2) letter distribution that will be mailed from a central state-wide hospice agency to FCGs who have lost their patient to cancer in past year that explains the study and provides information on how to contact the study project manager if interested in participating; (3) study posting on community social media websites such as Facebook; and (4) study registration on ResearchMatch, an on-line research volunteer registry. Strategies to recruit racially, ethnically, socioeconomically, and geographically diverse FCGs include ensuring broad representation of hospice sites that are in both urban and more remote rural areas that serve diverse populations (53). Further, filters will be applied that target racially and ethnically diverse populations when using the on-line research volunteer registry to increase a broader more representative group despite the convenience sampling methodology (53).

Interested potential participants complete informed consent prior to enrollment. All forms, surveys, and meditation modules are hosted on the secure HIPAA-compliant online platform, Qualtrics. To ensure eligibility, participants answer questions relative to the study eligibility criteria. Once confirmed, participants are assigned a unique three-digit study ID number for de-identification purposes and receive a link to complete the baseline Qualtrics surveys. Following completion of the Time 1 surveys, participants receive a link to the intervention modules, with orientation to the study. Study staff are available throughout the six-week period to conduct weekly check-ins, troubleshoot technical issues, and offer additional support to the FCG if needed.

At the end of the 6-week time, participants receive access to the Time 2 surveys, and again 6 weeks later at week 12 (Time 3). Participants receive a small gift card acknowledgement for their participation and continued access to the modules if desired. Fifteen participants will be interviewed using HIPAA compliant video-conferencing software to glean further information regarding the acceptability and feasibility parameters, and to identify any recommendations for wider-scale testing. Participants who consent to an interview will be contacted following completion of the week 12 surveys to schedule a date/time. The demographic and socioeconomic background of FCGs will be reviewed, and a representative sample will be invited to participate in the online interviews. With consent, these interviews will be recorded for data analysis purposes. The interview guide reflects questions that elicit important information relative to the NBHM experience, including both satisfaction and challenges in relation to acceptability and feasibility that can be used to strengthen the intervention on a larger scale.

### 2.4 Intervention

Six modules integrating meditative practices with nature imagery were developed for online delivery, drawing on the strength of our team’s previous meditation and nature intervention work (54, 55), with input from a meditation therapist professional. Each module is framed and anchored to the same core constructs: attention restoration, grief awareness and processing, psychological wellbeing and promotion of QOL. Once the content was developed, each 15-to-20-min module was audio-recorded by the technical team with the verbiage delivered by an experienced meditation provider. A brief version (~10 min) of the same content was also recorded using a simulated voice for those with less time to commit to the practices or who might prefer an alternative voice. After the modules were recorded with both voice versions, all were alpha tested by research team members to identify overall functionality and to identify any problems needing addressment prior to use with actual bereaved CGs. All modules will be available to the enrolled CGs via a private online link through Qualtrics. The study project manager will send each participant the access link to all modules once baseline data is collected. The intervention modules include orientation to the content, clear guided instruction, and motivational content to support the establishment of a regular practice. The meditations are all designed to be done seated comfortably in a chair. Participants will be encouraged to wear headphones and eye masks to focus on the auditory experience.

### 2.5 Fidelity

Standard NIH recommendations (dose, training, delivery, receipt, enactment) for socio-behavioral research will be used for intervention fidelity (56) and are depicted as following. Participants are asked to commit to completing a minimum of 4 modules over the 6-week period. The privately accessible website will record when an FCG accesses any module and the duration of use to establish doses received by individual participants. Because the intervention modules are pre-recorded and all training content on the web is uniform, there is no deviation in the provision of the intervention across participants. The intervention site which is accessible to study participants, records access and time spent with modules. Weekly emails, texts or calls (based on preference), using a toll-free access number during weeks 1–6, will provide reminders to engage with the nature meditations and to query if any technology assistance is needed. At week 12, meditation module access during the follow-up phase between weeks 7 to 12 will be monitored to assess sustained use of the intervention. All study staff will receive standardized training with active feedback prior to engaging with study participants. Additionally, their training will equip them to be available to provide technical troubleshooting assistance for participants.

### 2.6 Measures

#### 2.6.1 Demographic/health characteristics

At baseline (Time 1), a sociodemographic survey will collect information on sex, age, race, ethnicity, education, socioeconomic situation, relationship status, and time since death of the patient from cancer (in months). Information about comorbid conditions as a proxy of FCG health will be assessed using the standardized and validated 21-item Bayliss Comorbidity Index to identify number and types of chronic conditions and their level of interference in daily activities (57).

#### 2.6.2 Acceptability/feasibility

At 12 weeks (Time 3) the self-reported 4-item Acceptability of Intervention Measure (AIM) (58) and the 4-item Feasibility of Intervention Measure (FIM) will be used (58). The AIM evaluates acceptability with questions about satisfaction and appeal of the intervention. The FIM evaluates feasibility with questions about benefits, ease of use, and manageability of the intervention. The number of FCGs consented vs. completed, and the number of times and weeks using the intervention will also be assessed.

#### 2.6.3 Grief

The PG-13-Revised Prolonged Grief (PG-13-R) scale, a validated 13-item self-report measure aimed to assess a protracted grief experience while also providing an indicator of the bereavement process, will be used (19) to assess grief symptoms at the three time points.

#### 2.6.4 Directed attention

The Attention Function Index (AFI), a 16-item self-rating scale that measures perceived effectiveness in daily tasks that require cognitive function, an strong indicator of directed attention capacity. The AFI has demonstrated consistent reliability in both medical groups and among healthy adults (59) and will be used to assess directed attention at all three time points.

#### 2.6.5 Quality of life

QOL domains, including physical, social and psychological functioning and health perceptions, will be measured using the PROMIS-29 (60, 61) at all three time points. The PROMIS is a collection of short-form profile instruments (social, psychological, and physical function, pain, fatigue, sleep disturbance, health satisfaction) (62).

#### 2.6.6 Emotional symptoms

Depressive and anxiety symptoms will be assessed with the two respective PROMIS-29 short-forms (62) at all three time points.

### 2.7 Data analysis

#### 2.7.1 Power

Consistent with the analytic model (linear mixed-effect model, LMM) for estimating the change in the primary endpoint, QOL, a simulation-based approach (63) to estimate power to detect effect sizes (ESs) ranging from 0.2 (small) to 0.4 (moderate) is used. Because there is no existing literature on the within-participant correlation ( ρ ) of the outcome over time, three scenarios for ρ (0.3, 0.4, 0.5) are used with 1,000 simulations for each of the three ESs (0.2, 0.3, 0.4) and the baseline sample sizes ranging from 40 to 70 for each simulation. For the power of 0.80 and *α* = 0.05, an initial sample size of 42 FCGs is adequate to detect an ES of 0.4 if ρ= 0.4 and 55 FCGs are needed if ρ= 0.3; 51 FCGs will be adequate to detect an ES of 0.3 if ρ= 0.5 and 65 FCGs are needed if ρ= 0.4; and more than 70 FCGs will be needed to detect an ES of 0.2 across all scenarios for ρ. To be conservative and within the budget, and assuming a 20% attrition rate by week 12, a total of 70 FCGs will be recruited.

Basic summary statistics for demographic characteristics and co-morbid conditions in FCG will be calculated. Because the study involves repeated measurements on the same FCG, basic summary statistics will be generated for each of the three time points. All analytic procedures envisioned for this exploratory project are consistent with general best practices, including recommendations made by the National Research Council panel on the prevention and treatment of missing data in clinical trials (64).

Any technology issues that emerge will be meticulously monitored and tracked. To estimate the change in FCGs’ outcomes across time, an LMM for continuous outcomes will be used. The results will be presented in both the original and standardized scales with estimated confidence intervals and no statistical hypothesis testing will be performed. The random effects will account for the correlation/covariance within the FCGs arising from the repeated measurements on each FCG. Further exploratory analyses will be conducted to assess the potential heterogeneity of the longitudinal profile of the outcomes by FCGs’ baseline characteristics (baseline QOL, age, gender, and relationship). Operationally, this heterogeneity will be assessed through interactions between time and any of the FCGs’ baseline characteristics. Estimates from these analyses will provide directional effects for a later larger-scale study.

Acceptability will be assessed at two levels: (1) among FCGs approached, how many consent to participate; and (2) among participants, how many complete the intervention. For feasibility we establish the following minimum parameters: FCGs must complete a minimum 4 modules during the 6-week period. The study will use latent class analysis to group FCGs into several clusters based on AIM and FIM measures and examine whether FCGs’ demographic and outcome measures differ by cluster. This analysis will inform the relationship between acceptability and feasibility and the outcomes. The web-tracking data will be used to examine the time of day and duration each FCG uses each module. An exploratory analysis of the association between timing and duration of use and endpoints will be performed to estimate a response curve and identify the minimum intervention use associated with meaningful improvement and the potential intervention saturation point (i.e., the effect plateaued).

#### 2.7.2 Interview data

The semi-structured interviews will provide rich contextual data on perceived acceptability and feasibility of the NBHM program to support bereavement. The sample will be carefully selected to ensure a broad range of perceptions in relation to socio-demographic background and user data (low to high engagement). The audio recordings will be transcribed verbatim. Transcriptions will be assessed for accuracy by verifying the text against the audio recordings. Similar to the team’s previous studies with hospice FCGs (65, 66), two trained investigators will first organize the results and identify major content categories independently, then compare and discuss until a consensus is achieved. Data summaries from each semi-structured interview will be assessed against the interview questions and then compared, with targeted attention to collective similarities, to inform content category development.

## 3 Discussion

This study protocol paper describes the development and proposed assessment of a novel intervention tailored for bereaved FCGs of cancer patients. The discussion highlights the intervention’s relevance, addresses key gaps in the bereavement care literature, and positions NBHM within the broader context of public health, aging, and FCG well-being.

The proposed NBHM intervention is distinct in critical ways. First, it focuses explicitly on the post-death period, a time when cancer FCGs are vulnerable to complicated grief. While many existing psychosocial interventions target caregivers during the active phase of patient illness or at the end of life, structured follow-up care for FCGs following the death of a loved one remains inconsistent and, in some settings, virtually nonexistent (67). By directing attention to this underserved group, the NBHM intervention expands the continuum of FCG support beyond death, meeting caregivers during a period of role identity transition and major life changes (7, 24, 68). Second, the NBHM model operationalizes principles from mindfulness meditation, grief theory, and environmental psychology research to build a multifaceted, low-barrier intervention (46, 69, 70). Unlike modalities that require clinical facilitation and sustained participant engagement, NBHM can be accessed at home by those with internet access. Thus, there is increased potential for reaching FCG populations in underserved geographic areas, those with limited income or mobility, and those who may not self-identify as needing “formal” mental health care. The integration of nature-based elements (e.g., auditory simulation of landscapes, seasonal metaphors) allows users to build restorative mental environments, even when physical access to nature is limited, a concern for bereaved FCGs managing fatigue, chronic conditions, inclement weather, and/or limited transportation (45).

The study builds on a foundation of preliminary work involving meditative interventions with FCGs allowing for reflective adaptation of delivery methods and content preferences (54, 55). These adaptations were grounded in feedback that highlighted the need for short, calming, and emotionally attuned practices that can be revisited without pressure. In this way, the NBHM promotes autonomy and personalization - qualities that may increase engagement with an intervention aimed at a highly vulnerable population.

Implications for public health: In addition to its clinical relevance, the NBHM intervention addresses a significant public health priority. As the aging population grows and more patients die at home given personal preferences (1), the impact on informal caregivers will intensify. Ongoing higher grief among bereaved individuals has been linked to increased healthcare utilization, lost productivity, and worsened long-term physical and mental health outcomes (13, 14). An intervention that is accessible, scalable, and aligned with caregiver needs may have future potential to relieve strain on healthcare systems. Further, it is cost-effective, enabling access potentially for a broad cross-section of bereaved friend/family cancer FCGs given time, travel, mobility issues, and weather constraints that prevent use of more traditional delivery approaches. Finally, if successful, the NBHM intervention could potentially be integrated into existing hospice bereavement programs or community resource centers with minimal cost or infrastructure.

## 4 Limitations

The study relies on a convenience sample of bereaved FCGs and thus is subject to self-selection bias. Further, the lack of a control group, reliance on self-report measures, and restriction to English speaking participants only also reduce generalizability. As a feasibility and acceptability study, the goal is to assess whether the intervention is usable, satisfactory, and capable of producing promising effect sizes. Further, while the intervention is designed to be low-technology, variations in digital access, personal comfort levels, and adherence may still arise. Strategies such as weekly reminders and optional technical support are intended to buffer this risk. However, given the population digital technology divide (71), the voices of potential FCGs who are technologically less literate or who do not have access to a computer will not be represented in the study (71).

## 5 Conclusion

The NBHM intervention testing will assess acceptability and feasibility to validate the appropriateness for future work. Important information from cancer FCGs will be generated relative to their experiences with the nature meditations that will be useful towards refining the intervention prior to larger scale testing in a randomized control trial format. While the intervention does not attempt to replace grief counseling or other professional mental health support services, the NBHM may potentially present a complementary self-care resource that can be used privately or alongside other forms of care. With further testing, the NBHM may also offer positive benefits by providing hospice health professionals with an additional resource to support adaptation among bereaved FCGs of patients who died from cancer.

## Data Availability

The original contributions presented in the study are included in the article/supplementary material, further inquiries can be directed to the corresponding author/s.
